# Environmental and Public Health Implications of Water Reuse: Antibiotics, Antibiotic Resistant Bacteria, and Antibiotic Resistance Genes

**DOI:** 10.3390/antibiotics2030367

**Published:** 2013-07-31

**Authors:** Pei-Ying Hong, Nada Al-Jassim, Mohd Ikram Ansari, Roderick I. Mackie

**Affiliations:** 1 King Abdullah University of Science and Technology (KAUST), Environmental Science and Engineering, Water Desalination and Reuse Center, Thuwal 23955-6900, Saudi Arabia; E-Mails: nada.aljassim@kaust.edu.sa (N.A.-J.); mohd.ansari@kaust.edu.sa (M.I.A.); 2 Department of Animal Sciences, University of Illinois at Urbana-Champaign, Urbana, IL 61801, USA; 3 Institute of Genomic Biology, University of Illinois at Urbana-Champaign, Urbana, IL 61801, USA

**Keywords:** antibiotics, water reuse, antibiotic resistant bacteria, municipal wastewater, livestock manure, manure-applied soil

## Abstract

Water scarcity is a global problem, and is particularly acute in certain regions like Africa, the Middle East, as well as the western states of America. A breakdown on water usage revealed that 70% of freshwater supplies are used for agricultural irrigation. The use of reclaimed water as an alternative water source for agricultural irrigation would greatly alleviate the demand on freshwater sources. This paradigm shift is gaining momentum in several water scarce countries like Saudi Arabia. However, microbial problems associated with reclaimed water may hinder the use of reclaimed water for agricultural irrigation. Of particular concern is that the occurrence of antibiotic residues in the reclaimed water can select for antibiotic resistance genes among the microbial community. Antibiotic resistance genes can be associated with mobile genetic elements, which in turn allow a promiscuous transfer of resistance traits from one bacterium to another. Together with the pathogens that are present in the reclaimed water, antibiotic resistant bacteria can potentially exchange mobile genetic elements to create the “perfect microbial storm”. Given the significance of this issue, a deeper understanding of the occurrence of antibiotics in reclaimed water, and their potential influence on the selection of resistant microorganisms would be essential. In this review paper, we collated literature over the past two decades to determine the occurrence of antibiotics in municipal wastewater and livestock manure. We then discuss how these antibiotic resistant bacteria may impose a potential microbial risk to the environment and public health, and the knowledge gaps that would have to be addressed in future studies. Overall, the collation of the literature in wastewater treatment and agriculture serves to frame and identify potential concerns with respect to antibiotics, antibiotic resistant bacteria, and antibiotic resistance genes in reclaimed water.

## 1. Introduction

Water scarcity is a global issue, and is a result of economic and physical constraints. A survey by the International Water Management Institute listed certain regions like Africa, the Middle East, as well as the western states of America as water stressed areas [1]. A breakdown on the freshwater consumptive use in the United States (U.S.) showed that agricultural irrigation accounts for up to 81% of the total daily usage, while consumptive usage by domestic households only accounts for 6% of the total daily usage [2]. Given that applications like agricultural and landscaping irrigation do not require high quality water supply, water reuse has become an attractive option for conserving and extending available water supply.

Reclaimed water is technically defined as municipal wastewater that has gone through various treatment processes, and should only be used when the treated water quality falls in line with specific water quality criteria. For instance, the guidelines of U.S. Environmental Protection Agency (USEPA) stated that municipal wastewater would need to undergo secondary and/or tertiary treatment to achieve a considerable reduction in the organic and inorganic constituents, as measured based on biochemical oxygen demand (BOD) and chemical oxygen demand (COD). In addition, appropriate treatment has to be utilized to achieve no detectable fecal coliform or less than 200 fecal coliforms/100 mL in the reclaimed water prior to surface irrigation on any food crop or orchards and vineyards, respectively [3].

As municipal wastewater is a relatively stable and reliable flow of water that is rich in nitrogen and phosphorus content arising from the fecal contents, it is often deemed as an attractive source of water for agricultural irrigation. Livestock production farms in U.S. are no exception as they generally rely on animal manure as a nitrogen and phosphorus rich fertilizer for their agricultural crops. In most instances, the animal manure is first retained in a manure pit or lagoon over a period of time, before application on the agricultural field. Application can be applied by spraying over crops or through a direct injection into the soil at a 20 cm depth [4].

Although the use of municipal wastewater as an alternative water source is a highly attractive option, there is a need to understand the microbial risks arising from antibiotic residues, the antibiotic resistant bacteria, and their associated resistance genes. This is particularly complicated when antibiotics are increasingly consumed for disease treatment in humans and livestock, and as prophylaxis and growth promoters for the latter. The European Surveillance of Antimicrobial Consumption (ESAC) project collected data on antibiotic use for the period 1997–2001, and determined that the median national hospital antibiotic consumption in Europe was 2.1 DDD/1,000 inhabitants/day [5]. Defined daily dose (DDD) is the assumed average maintenance dose per day for a drug used for its main indication in adults [6]. Assuming an average dose of 750 mg for most of the examined antibiotics, that would amount to approximately 1,067 kg of antibiotics consumed per day in Europe. Surprisingly, hospital care consumption only accounts up to 17.8% of the total antibiotic consumption in Europe [5], while majority of the antibiotics are consumed in normal households.

In the same manner, livestock production utilizes equal, if not more antibiotics than in the human population. The Union of Concerned Scientists reported that approximately 11 million kg of antibiotics were used non-therapeutically in the swine, poultry and cattle production industries [7]. This accounts for more than 50% of the total antibiotics consumption in U.S. per annum [7]. The rampant use of antibiotics among both human and animal populations clearly suggests that large amounts of antibiotics would end up in municipal WWTPs and in animal manure. Indeed, environmental reservoirs are increasingly viewed as one of the major hotspots for various microorganisms to gain antibiotic resistance. As such, the presence of antibiotics would have to be examined if wastewater is to be reused.

In the following review, we seek to provide a comprehensive overview on the types and concentration of antibiotics that are detected in municipal wastewater and livestock manure. We then collate the abundant, various antibiotic resistance genes that are present in municipal wastewaters and treated effluent, as well as their abundance in animal waste lagoon and manure-applied soils. Finally, we discuss the significance of these contaminants pertaining to the use of reclaimed water to alleviate water scarcity.

## 2. Antibiotics in Municipal Effluent

In 2002, an estimated figure on the antibiotics consumed annually worldwide was between 100 million and 200 million kg of antibiotics [8]. A collation on the antibiotic prescription in a Portugese medical study revealed that the most frequently prescribed antibiotics were penicillin (47%), macrolides (16%), quinolones (15%), cephalosporins (12%), sulfonamides (5%) and tetracyclines (2%) [9,10]. In British Columbia, a similar trend in the usage of different classes of antibiotics was also observed. Beta-lactams like penicillin and cephalosporins were most commonly consumed in British Columbia, followed by tetracyclines, macrolides, sulfonamides and fluoroquinolones [11]. The mechanisms of each different class of antibiotics have already been extensively reviewed, and will not be included here. Readers can refer to the review paper by Kohanski to understand the mechanisms underlying the antibiotics mode of action against bacteria [12]. Most of these antibiotics are excreted from the human body, and are excreted as the parent compound in the feces or urine, which in turn ends up in the municipal wastewater treatment plant.

In a conventional municipal wastewater treatment process, municipal wastewater is first screened and clarified to remove large particulates and suspended particulates, respectively, prior to the biological treatment process in activated sludge. The treated water after the biological process is then pumped into a secondary clarifier to further remove suspended particulates and organic constituents. Typically, the treated water at this point is termed as the secondary treated effluent, and may undergo post-treatment processes (e.g., sand filtration, membrane filtration or disinfection). The tertiary treated water then gets reused for various purposes such as agricultural irrigation. Based on the treatment schematics, the quality of the effluent is generally anticipated to meet the stipulated guidelines of 5–30 mg/L of BOD [3], and is appropriate for irrigation on food and nonfood crops.

With the increasing use of municipal wastewater for various types of reclaimed water purposes, guidelines are also formulated to provide guidance [13] on the minimal achievable quality required for the reclaimed water. However, these guidelines do not include the minimal concentration of antibiotics residues and abundance of resistant bacteria that are allowed in reclaimed water. It is gradually recognized that antibiotics are emerging contaminants that can result in a “perfect microbial storm”, defined as a phenomenon where novel microbial threats emerge with elevated frequency and that can create an environment that allows infectious diseases to emerge and become rooted in society [14]. Clearly, the current guideline is not updated with emerging contaminants such as antibiotics residues and antibiotics resistant bacteria.

The removal efficiency of various types of antibiotics was recently reviewed [15]. Generally, removal is achieved via chemical treatment and/or bio-adsorption onto particulates and subsequently physical separation from municipal wastewater after sedimentation in the screening chambers and clarifiers. Therefore, the removal efficiency is highly dependent on the hydrophobicity and sorption capability of the antibiotics. In contrast, biological degradation is deemed to be relatively less effective in removing antibiotics from the bulk of the municipal wastewater. Based on this conventional treatment scheme, the abundance of antimicrobials in municipal wastewater treatment in Croatia was found to range from 2–20 μg/L [16]. Antibiotics like sulfapyridine (sulfonamides), azithromycin (macrolide) and erythromycin (macrolide) were not removed effectively. In particular, trimethoprim (dihydrofolate reductase inhibitor) was not sufficiently removed by the treatment process. This low removal efficiency can perhaps be explained by the low sorption potential of most sulfonamides (*i.e.*, logK_ow_ < 2.5) and medium sorption potential for macrolides (*i.e.*, 2.5 < logK_ow_ < 4) [16,17]. However in certain instances, high removal rates of sulfonamides like sulfamethoxazole and quinolones like norfloxacin and ciprofloxacin can still be achieved. Nevertheless, the detected abundance of these antibiotics residues in the secondary effluent still remained high, ranging at concentration of 119–544 ng/L, 24–175 ng/L and 11–168 ng/L, respectively, in the secondary effluent [16].

Alternatively, coupling the conventional treatment process with additional tertiary treatment like membrane filtration can further enhance the removal efficiency of antibiotics. The membrane filtration process achieves liquid-solid separation based on the pore sizes of the attached membrane. Given the small molecular size of antibiotics, microfiltration and ultrafiltration membranes that are connected to the bioreactors would not be able to provide the appropriate size exclusion to remove antibiotics from the municipal wastewater. Instead, a nanofiltration or reverse osmosis membrane would be more appropriate for the removal of antibiotics [18], although a full removal rate does not seem to be attainable for certain antibiotics even when a reverse osmosis membrane is used [19]. Moreover, a membrane bioreactor connected to nanofiltration or reverse osmosis membranes would require a higher operating pressure and therefore incur a higher energy landscape and operating costs. Considering that biofouling and organic fouling are both ubiquitous events on the membrane, these biofilm matrixes can be viewed as an additional barrier that will serve to improve the antibiotic removal efficiency. As such, most of the existing treatment plants which opt for membrane filtration as an additional tertiary treatment step would use microfiltration and ultrafiltration membranes to achieve a reasonably good effluent quality in consideration of the cost and benefits.

An additional advantage of the membrane bioreactor is the provision of high solid retention time within the bioreactor. Retentate or reject stream contains antibiotics which did not pass through the membrane, and would be recycled back to the bioreactor. Membrane filtration therefore provides a longer retention time for the antibiotics, and would provide more reaction time for the subsequent breakdown and hydrolysis of the antibiotics through biological degradation. To illustrate, the removal efficiency of certain antibiotics like trimethoprim and macrolides (e.g., erythromycin and clarithromycin) were significantly reduced by up to 90% at a solid retention time period of 60–80 days [20]. 

Anaerobic membrane processes would also be beneficial for its use to obtain reclaimed water suitable for agricultural irrigation, as nitrogen and phosphorus are retained during an anaerobic biological process while a conventional aerobic municipal wastewater treatment process would remove up to 70% of the nitrogen and 50% of the phosphorus content [21]. Furthermore, anaerobic membrane processes demonstrate the potential to create an energy neutral or positive landscape. However, there is a need to determine the persistence of pathogens and antibiotic resistance genes in the municipal water after going through anaerobic treatment processes. The persistence of plasmids in the different stages of municipal wastewater treatment plant was recently examined [22,23], and contradictory observations were reported. Merlin *et al.* [22] determined that the copy number of pB10 plasmids to DH5 chromosomal DNA increased over time under anaerobic conditions, and suggest an increase in the occurrence of plasmid transfer. Rysz and coworkers [23], however, noted that a higher rate of antibiotic resistance gene loss was observed in *E. coli* under anaerobic fermentation conditions than under aerobic conditions.

## 3. Antibiotic Resistant Bacteria and Associated Genes in Municipal Effluent

It is known that the human gut contains bacteria that are intrinsically resistant to antibiotics [24], and thus, many of the bacteria that enter into the treatment system may already be resistant to antibiotics. Furthermore, the existing municipal wastewater treatment design is unable to remove antibiotic resistant bacteria and their associated resistance genes entirely. The activated sludge is therefore exposed to antibiotic residues that may impose a selection for resistant bacteria. The confluence of gut-associated antibiotic resistant bacteria, remnant antibiotic residues and a rich diversity of activated sludge microbial consortium would suggest that the biological treatment process provides favorable environment for mobile genetic elements to be transferred among the microbial communities [15,25].

Past studies have isolated microorganisms from the activated sludge, and examined them for the presence of mobile genetic elements. These studies have demonstrated that many of the antibiotic resistance genes are associated with mobile genetic elements [26,27], which in turn suggest that mechanisms to transfer the genes from one bacterium to another are present. Mobile genetic elements can be transferred in three modes: (i) transformation, a process by which free DNA is incorporated into a competent cell and brings about genetic change in the recipient; (ii) conjugation, a process of genetic transfer that involves cell-to-cell contact; and (iii) transduction, a process by which DNA is transferred by bacteriophage.

At the same time, the microbial community in the activated sludge remains exposed to the antibiotics residues, and can develop resistance against these antibiotics. A study found that tetracycline resistance genes were present in the activated sludge sampled from 15 WWTPs at different geographical locations. In particular, *tetG* genes were present in the highest concentration of 1.75 × 10^–2^ ± 2.43 × 10^−3^ copies per copy of 16S rRNA genes compared to the other monitored tetracycline resistance genes (e.g., *tetA*, *tetB*, *tetC*, *tetD*, *tetE*, *tetG*, *tetK*, *tetL*, *tetM*, *tetO*, *tetP*, *tetS*, *tetX*). This high abundance of *tetG* genes is followed by *tetC*, *tetA* and *tetS* genes [28], further suggesting that the biological unit is a hot spot for antibiotic resistance genes.

Zhang and coworkers demonstrated that the prevalence of antibiotic resistance in *Acinetobacter* isolates increased along the wastewater treatment flow path. Specifically, the number of *Acinetobacter* isolates that are resistant to three or more antibiotics significantly increased from 33%–72.4% from the influent to effluent samples. However, upon discharge into the river environment, the prevalence of resistant *Acinetobacter* isolates decreased further downstream of the discharge point (56.5%) [29], suggesting a natural die-off or loss of resistance among these isolates when exposed to the indigenous microbial community in the environment. In an earlier study that was performed to isolate *Acinetobacter* spp., it was noted that the prevalence of antimicrobial resistance among the 442 isolates, showed no significant difference in antimicrobial resistance for most of the antibiotics tested, except for cefotaxime and nalidixic acid. The prevalence of *Acinetobacter* spp. that was resistant to cefotaxime decreased from 16.9%–5% in the treated effluent than in raw influent, while the prevalence of isolates that was resistant to nalidixic acid increased from 1.5%–10% [30]. This observation suggests a loss or gain of certain mobile traits that are associated with the different antibiotic resistance genes. Alternatively, the presence of antibiotics in the activated sludge process provides a selective pressure and favorable conditions for horizontal gene transfer of antibiotic resistance genes.

Besides *Acinetobacter* spp., viable enterococci and *Enterobacteriaceae* were isolated from municipal wastewater at different stages of the treatment process, and showed that many of the enterococci [9] and *Enterobacteriaceae* [31] were resistant to more than one antibiotic. The collective proportion of *Escherichia*, *Shigella* and *Klebsiella* spp. that were resistant to more than two antibiotics increased from an average 11.1% in the raw wastewater to 21.4% in the treated wastewater [31]. Similarly, the collective proportion of these *Enterobacteriaceae* which were resistant to three antibiotics increased from 5.5%–14.1% in the treated wastewater [31]. This observation further suggested that the conventional municipal wastewater treatment scheme does not effectively remove viable *Enterobacteriaceae* that are resistant to antibiotics.

To counter this problem, most wastewater treatment plants opt for chlorination of effluent in an attempt to disinfect any potential viable microorganisms that may be present. The typical chlorine dosage required to achieve total coliform disinfection based on a 60 min contact time ranged from around 2.5–20 mg/L to meet a total coliform concentration of 23–200 MPN/100 mL [13]. Although chlorination is able to achieve approximately 4–6 log removal or destruction of total coliforms [13], studies have shown that a significant portion of the antibiotic resistant fecal-associated bacteria remain viable. Huang *et al.* [32] examined different doses of chlorination, and the subsequent impact on the inactivation and regrowth of different types of antibiotic resistant bacteria. The study concluded that high dosages of chlorination can especially enhance the recovery of chloramphenicol-resistant bacteria, while low dosages of chlorination tend to result in an increased regrowth of chloramphenicol, ampicillin and penicillin-resistant bacteria.

Although it remains extremely useful to examine selective bacterial populations (e.g., *Acinetobacter* and *Enterobacteriaceae*) and their resistance traits, most of these approaches are culture-dependent and may not provide a comprehensive depiction of the actual extent of resistance among the total microbial community. This is particularly significant considering that a majority of microorganisms (*i.e.*, up to 99.9%) in the environment cannot be cultivated [33,34,35]. For this, molecular-based approaches, particularly quantitative PCR, would be required to determine the abundances of antibiotic resistance genes that are removed by the wastewater treatment facilities, and to estimate the extent of resistance genes that are disseminated into the environment from the municipal wastewater effluent.

To examine antibiotic resistance genes, the commonly used molecular methods are heavily dependent on the design of appropriate primers that can be used to detect and quantify the abundances of these genes. There are challenges involved in using this approach, primarily when examining the beta-lactamase genes. This is due to the wide diversity of beta lactamase genes that have been identified thus far [36,37]. For example, Colomer-Lluch observed five clusters of *bla_CTX-M_* genes which did not share enough conserved sequence regions to allow the design of a primer that would target all *bla_CTX-M_* genes and associated variants [27]. This means that numerous primers would have to be designed to ensure a comprehensive coverage of different resistance genes.

Further search on published literature revealed that more studies are needed to collate the abundances of various types of antibiotics resistance genes present in the municipal wastewater influent and effluent (Table 1). Table 1 summarizes the quantitative measurements of antibiotic resistance genes (ARGs) that are present in raw and treated municipal wastewater. Currently, there are three ways to report the abundance of resistance genes. First, one can report the abundance of resistance genes by normalizing against the volume of sample that was processed and extracted for its genomic DNA. Second, abundance of resistance genes are normalized against the total copy of 16S rRNA genes that were detected in the sample. Third, one can report the abundance of resistance genes that was normalized against the mass of genomic DNA utilized for quantitative PCR. All three methods would give slightly different values and hence interpretations [38]. For example, the first method would be useful when performing quantitative microbial risk assessment as it provides an estimate of the abundance of resistance genes at which a subject is exposed to when a known amount of treated wastewater was reused. The second and third method provided an estimate of the ratio of resistance genes to the total biomass and bacteria, respectively, which are present in the sample. Normalization to 16S rRNA genes and biomass would account for minor variations in sample processing, such as differences in DNA extraction efficiency, and in turn allow appropriate comparisons to be made among different samples. Furthermore, an increase in the abundance of resistance genes against the 16S rRNA genes would indicate that while the wastewater treatment process is efficient in removing most bacteria, antibiotic resistance genes, possibly along with their resistant hosts, were not effectively reduced by the same treatment schematics. To illustrate, a conventional activated sludge process achieved less than 1 log reduction in the ratio of *tetA* and *tetC* genes against the amount of genomic DNA [26]. Similarly, different resistance genes have different removal efficiency by the same treatment schematic. Auerbach and coworkers reported a 3 log removal of *tetQ* per ng DNA while *tetG* has only 2 log removal in the same Wisconsin wastewater treatment plant [39].

**Table 1:** Abundance of antibiotic resistance genes that was present in untreated municipal wastewater and in treated effluent. Abundances were determined by quantitative PCR.

Antibiotics	Gene class	Abundance in raw water	Abundance in final discharge/impacted environment	Treatment procedure	Geographical location	Ref.
Beta-lactam						
	*bla_TEM-uni_*	10^6.15^/mL sample 10^2.51^/ng DNA 10^−4.74^/16S	10^5.61^/mL sample 10^2.38^/ng DNA 10^−3.64^/16S	AS + Cl	Massachusetts, USA	[38]
	*bla_M-1_*	10^5.35^/mL sample 10^1.10^/ng DNA	10^3.45^/mL sample 10^2.03^/ng DNA	AS + Cl	South Carolina, USA	[40]
	*bla_vim_*	10^−1.22^–10^1.26^/ng DNA	ND–10^2.2^/ng DNA	WWTP, not specified	Germany	[41]
	*ampC*	10^0.34^–10^2.66^/ng DNA	10^−0.27^–10^1.20^/ng DNA			
	*ampC*	NA	ND–10^1.84^/mL sample	AS + P and N	Sabadell, Spain	[42]
		NA	ND/mL sample	Galatone WWTP	Nardò, Italy	
		NA	ND/mL sample	AS + UF + RO	Torreele, Belgium	
	*bla_shv-5_*	NA	ND–10^2.06^/mL sample	AS + P and N	Sabadell, Spain	
		NA	ND/mL sample	Galatone WWTP	Nardò, Italy	
		NA	ND/mL sample	AS + UF + RO	Torreele, Belgium	
	*mecA*	NA	ND–10^2.75^/mL sample	AS + P and N	Sabadell, Spain	
		NA	ND/mL sample	Galatone WWTP	Nardò, Italy	
		NA	ND/mL sample	AS + UF + RO	Torreele, Belgium	
	*bla_TEM_*	10^4.4^/mL sample	10^3.8^/mL sample	Not specified	Barcelona, Spain	[27]
	*bla_CTX-M_*	10^3.3^/mL sample	10^2.1^/mL sample			
	*mecA*	10^3.7^/mL sample	10^2.2^/mL sample			
	*mecA*	10^1.7^/mL sample 10^−0.07^/ng DNA	10^0.7^/mL sample 10^−0.59^/ng DNA	AS + TF	Gothenburg, Sweden	[43]
Macrolide						
	*ermB*	NA	ND–10^4.42^/mL sample	AS + P and N	Sabadell, Spain	[42]
		NA	ND–10^3.13^/mL sample	Galatone WWTP	Nardò, Italy	
		NA	ND–10^3.28^/mL sample	AS + UF + RO	Torreele, Belgium	
	*ermB*	~10^9.70^/mL sample ~10^−2.30^/16S	~10^7.70^/mL sample ~10^−3.60^/16S	Water solids, aerobic digestor for approximately 3 months	Minnesota, USA	[44]
	*ermB*	~10^7.48^/mL sample ~10^−3.52^/16S	~10^2.3^–10^4.48^/mL sample ~ND–10^−3.82^/16S	Secondary effluent, activated sludge	Shafdan, Israel	[45]
	*ermF*	~10^7.78^/mL sample ~10^−3.22^/16S	~10^3.48^–10^5.3^/mL sample ~10^−3.05^–10^−2.52^/16S			
Tetracycline						
	*tetW*	10^5.37^−10^7.4^/mL sample ~10^−3.12^/16S	ND–10^3.63^/mL sample ND/16S	AS/OD/RBCs/MBR + UV/Cl	Michigan, USA	[46]
	*tetO*	10^5.51^–10^7.61^/mL sample ~10^−3.12^/16S	ND–10^3.96^/mL sample ND/16S			
	*tetQ*	10^7.2^–10^9^/mL sample 10^5.3^–10^6.8^/ng DNA	10^3.9^–10^6.2^/mL sample 10^3.7^-10^5.4^/ng DNA	AS/P and N/UV/Cl	Wisconsin, USA	[39]
	*tetG*	10^6.4^–10^7.8^/mL sample 10^4.5^–10^5.7^/ng DNA	10^4.2^–10^5.9^/mL sample 10^4^–10^5^/ng DNA			
	*tetC*	10^8.13^–10^8.3^/mL sample 10^5.45^–10^5.65^/ng DNA	ND–10^4.12^/mL sample ND–10^3.57^/ng DNA	AS + Cl	Hong Kong, China	[47]
	*tetA*	10^7.78^–10^8.2^/mL sample 10^5.09^–10^5.57^/ng DNA	ND–10^4.33^/mL sample ND–10^3.78^/ng DNA			
	*tetA*	10^7.7^/mL sample 10^5.55^/ng DNA	10^6.15^/mL sample 10^5.24^/ng DNA	AS	Nanjing, China	[26]
	*tetC*	10^7.91^/mL sample 10^5.76^/ng DNA	10^6.14^/mL sample 10^5.23^/ng DNA			
	*tetO*	NA	ND–10^4.44^/mL sample	AS + P and N	Sabadell, Spain	[42]
		NA	10^2.93^–10^4.58^/mL sample	Galatone WWTP	Nardò, Italy	
		NA	ND–10^5.02^/mL sample	AS + UF + RO	Torreele, Belgium	
	*tetA*	~10^8.85^/mL sample ~10^−3.15^/16S	~10^7.85^/mL sample ~10^−3.46^/16S	Water solids, aerobic digestor for approximately 3 months	Minnesota, USA	[44]
	*tetW*	~10^9.78^/mL sample ~10^−2.22^/16S	~10^7.95^/mL sample ~10^−3.35^/16S			
	*tetX*	~10^8.7^/mL sample ~10^−3.3^/16S	~10^9.48^/mL sample ~10^−1.82^/16S			
	*tetO*	NA	~10^3^, 10^1.7^, 10^2^/mL sample ~10^−2.3^, 10^−2.6^, 10^−2.7^/16S	Secondary effluent, chlorinated effluent, dechlorinated effluent	Western USA	[48]
	*tetW*	NA	~10^3.6^, 10^2.3^,10^2^/mL sample ~10^−1.7^, 10^−2^, 10^−2.7^/16S			
	*tetO*	~10^7.3^/mL sample ~10^−3.7^/16S	~ND−10^3^/mL sample ~ND–10^−4.4^/16S	Secondary effluent, activated sludge	Shafdan, Israel	[45]
	*tetM*	10^−3.87^–10^−2.42^/16S	NA	Rural domestic sewage treatment system, usually anaerobic digestor	Hangzhou, China	[49]
	*tetO*	10^−4.41^–10^−3.24^/16S				
	*tetQ*	10^−4.64^–10^−2.8^/16S				
	*tetW*	10^−3.16^–10^−2.03^/16S				
	*tetM*	~10^−2.70^–10^−2.30^/16S	NA	Urban WWTP, usually oxidation ditch or anaerobic oxic zones		
	*tetO*	~10^−3.00^–10^−2.70^/16S				
	*tetQ*	~10^−2.82^–10^−2.00^/16S				
	*tetW*	~10^−1.70^–10^−1.30^/16S				
Sulfonamide						
	*sul-I*	10^5.46^–10^7.54^/mL sample ~10^−3.4^/16S	10^4.37^–10^6.75^/mL sample ~10^−3.4^/16S	AS/OD/RBCs/MBR + UV/Cl	Michigan, USA	[46]
	*sul-I*	~10^6.4^/mL sample ~10^−1.52^/16S	~10^6.5^/mL sample ~10^−1.1^/16S	Not specified	Lausanne, Switzerland	[50]
	*sul-II*	~10^5.6^/mL sample ~10^−2.3^/16S	~10^5.5^/mL sample ~10^−2^/16S			
	*sul-I*	~10^8.90^/mL sample ~10^−3.10^/16S	~10^8^/mL sample ~10^−3.30^/16S	Water solids, aerobic digestor for approximately 3 months	Minnesota, USA	[44]
	*sul-I*	NA	~10^4.9^, 10^3.7^, 10^3.9^/mL sample ~10^−0.4^, 10^−0.6^, 10^−0.8^/16S	Secondary effluent, chlorinated effluent, dechlorinated effluent	Western USA	[48]
	*sul-II*	NA	~10^4.6^, 10^2^, 10^1.9^/mL sample ~10^−0.7^, 10^−2.3^, 10^−2.8^/16S			
	*sul-I*	~10^8.48^/mL sample ~10^−2.4^/16S	~10^4.78^–10^5.48^/mL sample ~10^−2.52^–10^−1.7^/16S	Secondary effluent, activated sludge	Shafdan, Israel	[45]
	*sul-II*	~10^8.30^/mL sample ~10^−2.7^/16S	~10^3.48^–10^4.88^/mL sample ~10^−3.3^–10^−2.4^/16S			
	*sul-I*	~10^−2.70^–10^−1.70^/16S	NA	Rural domestic sewage treatment system, usually anaerobic digestor	Hangzhou, China	[49]
	*sul-II*	~10^−2.52^–10^−1.15^/16S				
	*sul-I*	~10^−2.15^–10^−1.7^/16S	NA	Urban WWTP, usually oxidation ditch or anaerobic oxic zones		
	*sul-II*	~10^−2.00^–10^−1.70^/16S				
Others						
	*vanA*	<10^−0.09^/ng DNA	ND–10^−2^/ng DNA	WWTP, not specified	Germany	[41]
	*vanA*	NA	ND/mL sample	AS + P and N	Sabadell, Spain	[42]
		NA	ND/mL sample	Galatone WWTP	Nardò, Italy	
		NA	ND/mL sample	AS + UF + RO	Torreele, Belgium	

NA: not applicable, ND: not detected, AS: activated sludge, Cl: chlorine disinfection, WWTP: wastewater treatment plant, P and N: phosphorus and nitrogen removal (nutrient removal), UF: ultrafiltration, RO: reverse osmosis, TF: trickling filter, OD: oxidation ditch, RBCs: rotating biological contactors, MBR: membrane bioreactor, UV: ultraviolet disinfection.

Based on available literature, it was further observed that the removal efficacy for antibiotic resistance genes is dependent on the treatment scheme. For example, a conventional activated sludge process in Nanjing, China is able to reduce the concentration of *tetA* and *tetC* from approximately 10^8^ copies to 10^6^ copies per mL of sample [26]. Disinfecting the secondary effluent in Hong Kong was able to further reduce the abundance of *tetC* from 10^8.13^–10^4.12^ per mL of sample, and in most cases below detection limits (*i.e.*, more than 4 log removal). A similar reduction in the abundance of *tetA* was also observed (*i.e.*, more than 3 log removal) [47]. However, in other geographical locations like in Massachusetts and South Carolina, USA, a lower removal efficiency of the beta-lactamase genes was reported (Table 1). In both treatment plants, conventional activated sludge process coupled with chlorination achieved approximately 71.2% and 98.7% removal of *bla_TEM-uni_* and *bla_M-1_* genes, respectively [38,40].

Currently, most of the monitoring efforts are restricted to geographical locations like in USA and in Europe. Geographical locations like Middle East and Africa require a more comprehensive examination of antibiotic resistance genes in the municipal wastewater. This is especially important as these countries practice a large volume of wastewater reuse [1]. Based on Table 1, up to an average of approximately 10^4.67^, 10^6.92^, 10^8.18^, 10^6.96^ copies of beta-lactam, erythromycin, tetracycline and sulfonamide resistance genes, respectively, were discharged along with every milliliter of treated effluent into the environment from the WWTP. In Jeddah, Saudi Arabia, a total of 299,100 m^3^ and 73,300 m^3^ of treated wastewater is discharged and reused, respectively [51]. Collectively, these observations would mean that a large amount of antibiotics resistance genes are disseminated into the environment, and further supporting the concept that wastewater treatment plants are hotspots for antibiotics resistance genes dissemination and transfer [15,25].

## 4. Antibiotics in Livestock Production Farms

Antibiotic usage is not uniquely associated with the human population alone. Rampant antibiotics use occurs on most livestock production farms worldwide. In livestock production farms, antibiotics are commonly used for disease treatment and at sub-therapeutic levels for prophylaxis (*i.e.*, prevention of diseases) as well as for growth promotion. An extensive documented usage can be found in USA and in Europe where surveillance programs are in place to monitor the usage. In 2011, the U.S. reported sales and distribution of approximately 13.5 million kg of antimicrobial drugs. The predominant antimicrobial class that was used in livestock production was tetracyclines with an annual total of 5.6 million kg. This was followed by ionophores (4.1 million kg), penicillins (0.9 million kg), macrolides (0.6 million kg), sulfonamides (0.4 million kg), aminoglycosides (0.2 million kg), lincosamides (0.2 million kg) and cephalosporins (0.03 million kg). The remaining 1.5 million kg are a wide range of antimicrobial classes that are not typically independently reported, and include for example, amphenicols, fluoroquinolones, glycolipids, polypeptides, quinoxalines and streptogramins [52]. There are disadvantages in reporting antibiotic usage in terms of its sales and distribution as it gives no indication of the number of active doses administered on the animals. Therefore, Denmark and the Netherlands started reporting antimicrobial usage on livestock animals by Animal Daily dose (ADD) and Defined Daily Doses per animal year (DDD animal), respectively, which are both methods that are similar to that used by World Health Organization to monitor antibiotics use in humans.

Compared to their U.S. counterparts, EU has already banned all agricultural growth promoters since 2006. However, it was recently reported that even if a few animals were found to be sick, often the whole flock or herd would be treated (known as metaphylaxis) to prevent the disease spreading [53]. This practice may perhaps explain why Denmark still reported a total veterinary antimicrobial use of 107,900 kg in 2011. The main usage was reported in pigs (77%), cattle (14%), fur animals (4%), aquaculture (2%) and poultry industry (0.7%). Within the pig production industry, the major classes of antibiotics used were pleuromutilins (60%), tetracyclines (27%) and macrolides (26%). In the cattle industry, beta-lactams (e.g., penicillins), tetracyclines and macrolides were also used [54]. A similar trend was observed in the U.S. where pig production reported the highest use of antibiotics compared to the other livestock production activities.

Different phases of livestock production utilize varying amounts of antibiotics, and may therefore disseminate varying abundances of remnant antibiotics into the environment. To illustrate, the pig production comprises of four phases, namely gestation (*i.e.*, the phase at which sows become impregnated), farrowing (*i.e.*, the phase at which sows give birth), weaning (*i.e.*, the phase at which piglets are weaned off) and finishing (*i.e.*, the phase at which pigs are fattened to reach the targeted weight). The usage of antibiotics was estimated to account 70%–80% of feeds for pig starters (*i.e.*, feeds for piglets as they are weaned off from milk) and pig grower (*i.e.*, feeds to achieve maximum growth of pigs). Antibiotics were used in about 50%–60% of finisher feed, and 40%–50% of sow feeds, respectively [55]. As a result, a higher usage of antibiotics may be associated with farms specialized in pig nurseries than in finishing and farrowing farms. Furthermore, different phases of pig production may only be authorized to use certain types of antibiotics as prophylaxis or for treatment. The UK, for example, authorizes the use of amoxicillin in weaned piglets, while infections in fattening pigs are often prescribed with trimethoprim and sulfadiazine [53]. These management differences would mean that different phases of livestock production farms would most likely result in different extents of environmental impact.

The liquid manure from pig and cattle production farms is commonly utilized as fertilizers in the nearby agricultural fields, after stabilization in the waste lagoon. The waste lagoon functions as an aerobic or anoxic digestion treatment process, where organic matter gets degraded by microorganisms, and pathogens or other contaminants naturally decay after a prolonged retention time. Pei *et al.* examined the feasibility of aerobic and anaerobic treatment for the dairy lagoon water, and observed that while biological activities in both systems can effectively reduce the amount of antibiotics that are spiked into the system, both systems have varying removal efficiency in the different types of antibiotic resistance genes [56]. For example, given enough time (e.g., 140 day), an anaerobic system may be more effective in reducing the concentration of *sul-II* genes compared to the aerobic system. In contrast, an aerobic system is more effective in reducing *tetO* genes than anaerobic systems [56]. It is therefore important to first determine the baseline concentration and diversity of resistance genes that are present in the municipal wastewater or water sample before deciding on the type of biological treatment process to adopt. Particularly worth noting is that in all systems, there seems to be release of antibiotic resistance genes during the early days of biological treatment process (e.g., <30 day) and may actually facilitate the spread of antibiotic resistance genes if lagoon treatment is terminated too early prior to land application [56].

Fortunately, in most countries, application of the liquid manure coincides with crop cycles. Therefore, much of the manure is retained and stabilized for 6 months to 1 year prior application [4]. Generally, the liquid manure was applied onto the crop fields via three ways: surface application, surface application followed by incorporation, or by direct soil injection. One of the major shortcomings of surface application is that liquid manure along with the pathogens and antibiotic-resistant bacteria may be aerosolized into the air. Our previous study of bioaerosols sampled in the concentrated animal farms showed that fecal-associated bacterial populations can become airborne. The airborne bacterial populations were also positive for antibiotic resistance genes in their genomic DNA [57]. Furthermore, surface application would likely result in surface runoffs during a significant rainfall event, in turn facilitating the dissemination of antibiotics and the associated resistant bacteria into the environment.

Clearly, the current agricultural practice adopted by livestock production farms would mean that the water and soil bodies of close proximities to the livestock production farms often come into direct contact with antibiotics. The concentration of antibiotics residues that were detected in the manure-applied soil and in water was previously reviewed [4]. However, limited studies are available to provide insights to the degradation of these antibiotics in the natural environment. Among the commonly used veterinary antibiotics, tetracycline and its degradation products were detected using ELISA or liquid chromatography mass spectrometry (LC-MS) [58,59]. It was observed that chlortetracycline forms anhydro-chlortetracycline, while oxytetracycline forms β-apo-oxytetracycline and tetracycline forms anhydro-tetracycline, respectively [59]. The detection of these degradation products is likely due to the relative instability of the C6 benzylic hydroxyl group, which enable dehydration of the C-ring through the 6-OH group in the tetracycline molecules [58]. In a separate study, Aga and coworkers further detected the presence of several other degradation products (e.g., 4-epi-tetracycline and 4-epianhydrotetracycline), likely due to the C4 dimethylamino group in tetracyclines undergoing a reversible epimerization process [58]. These observations suggested that given a sufficient amount of time, degradation of antibiotics can be achieved, although it remains unknown if the degradation products would impose more toxicity or detrimental impacts than the parent compounds.

## 5. Antibiotic Resistant Bacteria and Associated Genes in Livestock Production Farms

Considering that some of the antibiotic classes used in livestock are also used in the human population, the use of these antibiotics in livestock production farms can be a concern if cross-resistance among bacterial pathogens was to occur. This is of even more concern when antibiotics are used at the sub-therapeutic level for growth promotion. Although antibiotics as a growth promoter has been used in practice since more than 50 years ago, the mode of action behind antibiotics as growth promoters still remain elusive until recently. Using a mouse model, Cho and coworkers observed that sub-therapeutic doses of antibiotics would alter the gut microbiota, resulting in an increase in the family Lachnospiraceae of phylum Firmicutes [60]. Although there were no significant changes in the total abundance of microbial cell counts, a bloom in the proportion of Firmicutes seem to coincide with previous observations, which noted that hosts with higher body-to-mass index seemed to have a higher relative abundance of Firmicutes to Bacteroidetes [61,62]. There was also a significant increase in the abundance of butyryl CoA transferase and formyltetrahydrofolate synthetase genes normalized against the total bacteria [60]. The increase in the abundance of both genes is associated with an increase in the butyrate and acetate. Both butyrate and acetate are short chain fatty acids which can provide additional energy to the host animal [63].

The phylum Firmicutes is one of the predominant enteric microbiota in most living hosts [64,65]. When excreted along with the rest of the fecal microbiota, some of the Firmicutes remain viable and survive stabilization process in the manure pit [66]. Other enteric bacteria like *E. coli*, enterococci and fecal streptococci also remained viable and were recovered from various types of animal manure [67,68,69]. There exists a possibility that the antibiotic resistant bacteria originating from the manure may survive and persist in the manure-applied soils. To illustrate, portions of soil samples from the agricultural crop fields associated with a swine production farm (Site C), as well as manure, were collected from Site C. The soil samples were resuspended into R2A and nutrient broth in 20 μg/mL of tetracycline and erythromycin to isolate bacterial colonies that were resistant to the two antibiotics (Mackie and Ekizoglu unpublished).

Nine isolates were isolated and further characterized for their 16S rRNA gene identity (Table 2). Among them, three isolates most likely originated from the animal manure, while the remaining six isolates originated from the soil. It was observed that the fecal-associated isolates generally exhibited higher minimum inhibitory concentration towards antibiotics than the soil isolates. In particular, isolate E3 shared a 99% BLASTN match to the 16S rRNA genes of *Staphylococcus simulans*, and exhibited resistance towards tetracycline (32 μg/mL) and erythromycin (256 μg/mL). To elucidate how isolates PM-ae, PM-3 and E3 would gain resistance to tetracycline, end-point PCR detection was utilized to detect for the presence of a wide spectrum of tetracycline resistance genes [70,71]. The tetracycline resistance gene *tetM* was positively amplified in PM-ae, but no correct amplification of the tested tetracycline resistance genes was observed in PM-3 and E3. The list of tetracycline resistance genes tested in this study was not exhaustive, and it is likely that resistance in strains PM-3 and E3 may be conferred by other gene classes that were not tested.

**Table 2:** Bacterial species isolated from soil samples. The minimum inhibitory concentrations (MIC) in tetracycline and erythromycin are listed. * denotes that the bacterial isolate exhibits either one or more of the examined motility traits (*i.e.*, swimming, swarming, and twitching). N.A. denotes no growth in that particular medium.

Isolate name	Origin	Nearest match based on 16S rRNA gene (Max identity %)	MIC (µg/mL)		Motility *			
			Tet	Erm	R2A	TYG	Nutrient	LB
PM-ae	Manure pit	*Enterococcus avium* (99%) JX185519	32	256	N.A.	-	-	-
PM-3	Manure pit	Uncultured bacterium clone (99%) JX185520	32	256	-	-	-	-
E3	Soil, 1 day after manure application	*Staphylococcus simulans* (99%) JX185521	32	256	-	-	-	-
CS6G3-1	Soil, 42 day after manure application	*Achromobacter* sp. (99%) JX185522	8	8	+	+	+	+
CS8G3-6	Soil, 42 day after manure application	*Dyella* sp. (99%) JX185523	8	4	+	+	+	+
CN3G2-10	Soil, 21 day after manure application	Uncultured *Flexibacter* sp. (99%) JX185524	16	16	-	-	-	-
ET13	Soil, 1 day after manure application	*Burkholderia cenocepacia* (99%) JX185525	4	16	+	+	+	+
ET8	Soil, 1 day after manure application	*Brevibacillus brevis* (99%) JX185526	<4	4	+	+	+	-
E20	Soil, 1 day after manure application	*Microbacterium takaoensis* (99%) JX185527	<4	4	+	+	+	+

**Figure 1:**
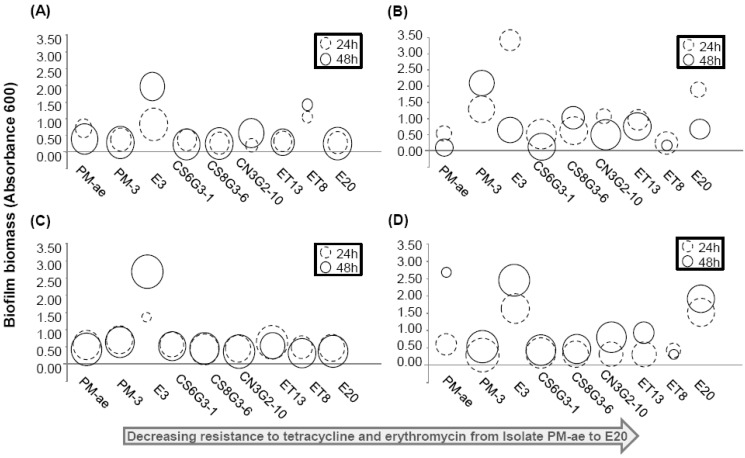
Bubble plots depicting the amount of attached biofilm formed by the individual bacterial isolate on the microtiter plate assay. The isolates were grown in (**A**) R2A, (**B**) TYG, (**C**) nutrient, and (**D**) Luria broth for 24 and 48 h. Isolates were arranged in order of their MICs towards tetracycline and erythromycin, in which PM-ae exhibited the highest MIC towards antibiotics, and vice versa for E20. The center of each bubble gives the value of attached biofilm biomass as quantified by crystal violet staining. The size of each bubble is a measure of cell density as quantified by OD_600_ measurement.

Besides possessing tetracycline resistance genes, a possible mechanism for these bacterial isolates to remain viable in the presence of antibiotics could be through biofilm formation [72]. Indeed, isolate E3 formed the highest amount of biofilm after 24 h and 48 h incubation, and in all four types of tested growth medium. The amount of biofilm produced by E3 is also up to 3.5-fold higher compared with the other isolates (Figure 1). It is therefore likely that tetracycline resistance in *S. simulans* strain E3 may be due to its enhanced biofilm formation relative to the other isolates (Figure 1), hence conferring a significant survival advantage to strain E3 when exposed to stressful environmental conditions and antimicrobials [73].

In addition to selecting for antibiotic resistant bacteria, the abundance of resistance genes was also found to correlate with the concentration of detected antibiotics residues [74]. Smith *et al.* also observed a weak but significant correlation between abundance of tetracycline resistance genes and the concentration of tetracycline residues that were detected in the waste lagoon [75], suggesting that a constant exposure of antibiotics would lead to selective pressure for resistance genes. However, contradictory results were observed in the groundwater samples, where the detection of resistance genes was often not associated with a detection of the corresponding antibiotics residues. The lack of correlation between antibiotic concentration and resistance gene abundance may be due to antibiotics and the resistance genes having a different fate and persistence in this environment. Antibiotics may be degraded to form intermediates that still select for resistance, hence explaining the persistence of antibiotic resistance genes. Upon degradation, the parent compounds may be present in low concentration that are not within the detection limits of most analytical equipment. Furthermore, most analytical methods focus on the detection of parent antibiotic compounds but not on the intermediate compounds formed during degradation. Therefore, a lack of correlation between the concentration of antibiotics and abundance of resistance genes does not disprove the hypothesis that low levels of antibiotics in the environment can drive the emergence of resistant strains in environment and in hospital settings. Instead, several epidemiological observations have concluded that the problem of antibiotic resistant bacteria is not necessarily linked to the persistence of antibiotic residues in the environment [76], but rather to the exposure to antibiotics and possibly other factors (e.g., presence of heavy metals) that may contribute to the selection of antibiotic resistance genes.

Table 3, Table 4 summarize the quantitative measurements of antibiotic resistance genes (ARGs) that are present in manure and in manure-applied soils, respectively. An extensive search on published literature revealed that many studies have noted positive detection of various resistance genes, particularly those conferring resistance to tetracycline, in both the lagoon waters and the manure-applied soils that were directly impacted by the livestock production farms (Table 3, Table 4). In particular, tetracycline resistance conferred by efflux pumps (*i.e.*, *tetA*, *tetB*, *tetC*, *tetG*, *tetH*, *tetL*, *tetZ*) and those conferred by ribosomal protection proteins (*i.e.*, *tetM*, *tetO*, *tetQ*, *tetW*) were present in high abundances per mass or volume of animal manure. Generally, stabilizing the manure in a lagoon achieved approximately less than 1 log reduction (*i.e.*, 77%) in the abundance of tetracycline resistance genes per mL of sample (Table 3). In contrast, chloramphenicol-resistance genes were below detection limit after stabilization. Nevertheless, groundwater that was directly adjacent to the manure pit remained susceptible to contamination events originating from the waste lagoon. Monitoring efforts have shown that water samples of close proximity to the waste lagoon were often contaminated with fecal-associated bacteria and antibiotics resistance genes that likely originated from the waste lagoon [77,78,79]. This indicates that while long term passive storage of lagoon is an economical solution to decrease the abundance of antibiotic resistance genes, care must be taken to maintain the integrity of the lagoon linings and to ensure that leachate from the lagoon does not compromise the groundwater quality.

**Table 3:** Abundance of antibiotic resistance genes present in livestock production lagoons and treated manure. Abundance was determined by quantitative PCR.

Antibiotics	Gene class	Abundance in the raw water	Abundance in the final discharge/impacted environment	Treatment procedure	Geographical location	Ref.
Macrolide						
	*ermA*	ND–10^−3.15^/16S	ND/16S	Lagoon Groundwater (Pig)	Illinois, USA	[80]
	*ermB*	10^−3.62^–10^−2^/16S	ND–10^−3.66^/16S			
	*ermC*	ND–10^−3.06^/16S	ND–10^−3.68^/16S			
Tetracycline						
	*tetW*	10^−3.2^–10^−3.0^/16S	10^−6.2^–10^−4^/16S	Lagoon Irrigation ditch (Dairy cattle)	Colorado, USA	[81]
	*tetO*	10^−3.9^–10^−3.5^/16S	ND–10^−5.2^/16S			
	*tetM*	10^−2.34^–10^−1.48^/16S	ND–10^−2.07^/16S	Lagoon Groundwater (Pig)	Illinois, USA	[82]
	*tetO*	10^−2.74^–10^−1.34^/16S	ND–10^−3.04^/16S			
	*tetQ*	10^−1.93^–10^−0.92^/16S	ND–10^−2.07^/16S			
	*tetW*	10^−2.53^–10^−1.33^/16S	ND–10^−2.35^/16S			
	*tetC*	10^−2.85^–10^−1.15^/16S	ND–10^−0.99^/16S			
	*tetH*	<10^−3.33^–10^−3.31^/16S	ND–10^−2.43^/16S			
	*tetZ*	<10^−4.22^–10^−2.46^/16S	ND–10^−2.55^/16S			
	*tetO*	10^4.1^–10^5.2^/mL sample	NA	Lagoon (Cattle)	Midwest, USA	[75]
	*tetW*	10^5.1^–10^5.5^/mL sample	NA			
	*tetQ*	10^4.9^–10^5.9^/mL sample	NA			
	*tetA* and *tetC*	10^6.0^–10^10^/g sample	10^6.8^–10^7.5^/g sample	Effluent from confinement houses Lagoon	North Carolina or Ohio, USA	[83]
	*tetG*	10^7.8^–10^8.8^/g sample	10^7.3^–10^8.4^/g sample			
	RPP (7 genes)	10^7.8^–10^9.2^/g sample	10^8.2^–10^8.7^/g sample			
	*tetM*	NA	~10^2.8^– 10^6.1^/mL sample ~10^−5.7^–10^−2.2^/16S	Cattle feedlot lagoons using different amount of antibiotics	Midwest, USA	[84]
	*tetO*		~10^2.7^–10^4.9^/mL sample ~10^−4.8^–10^−3^/16S			
	*tetQ*		~10^3.2^–10^5.5^/mL sample ~10^−5.7^–10^−3^/16S			
	*tetW*		~10^2^–10^4.5^/mL sample ~10^−4.8^–10^−3.1^/16S			
	*tetB*		~10^2^–10^3^/mL sample ~10^−5.6^–10^−5.3^/16S			
	*tetL*		~10^0.4^–10^1.8^/mL sample ~10^−6.7^–10^−5.2^/16S			
	*tetW*	NA	10^−2.4^–10^−1.8^/16S	Water solids, Lagoon (Beef, dairy)	USA	[85]
		NA	~10^−1.9^/16S	Water solids, Lagoon (swine)		
		NA	~10^−4.8^/16S	Water solids, Lagoon (chicken layer)		
	*tetQ*	10^−2.43^–10^−2.21^/16S 10^4.96^–10^5.22^/ng DNA	ND–10^−1.93^/16S ND–10^3.47^/ng DNA	Lagoon (Pig) Groundwater	Illinois, USA	[77]
	*tetZ*	10^−4.39^–10^−3.84^/16S 10^3.0^–10^3.59^/ng DNA	ND–10^−2.28^/16S ND–10^2.92^/ng DNA			
Sulfonamide						
	*sul–I*	10^−1.5^–10^−1.4^/16S	10^−2.6^–10^−1.6^/16S	Lagoon Irrigation ditch (Animal)	Colorado, USA	[81]
	*sul–II*	10^−4.3^–10^−3.9^/16S	ND/16S			
	*sul–I*	NA	~10^−2.5^–10^−2^/16S	Water solids, Lagoon (Beef, dairy)	USA	[85]
	*sul–II*	NA	~10^−1.4^–10^−1.1^/16S			
	*sul–I*	NA	~10^−1.4^/16S	Water solids, Lagoon (swine)		
	*sul–II*	NA	~10^−0.2^/16S			
	*sul–I*	NA	~10^−2.7^/16S	Water solids, Lagoon (chicken layer)		
	*sul–II*	NA	~10^−2.2^/16S			
Others						
	*cmlA*	10^4.8^–10^6.05^/mL sample 10^−2.15^–10^−1.49^/16S	NA	Lagoon (Swine feedlots)	Beijing, China	[74]
	*floR*	10^4.94^–10^6.05^/mL sample 10^−1.8^–10^−1.41^/16S				
	*fexA*	10^4.52^–10^6.21^/mL sample 10^−1.9^–10^−1.33^/16S				
	*cfr*	10^4.86^–10^6.1^/mL sample 10^−1.66^–10^−1.44^/16S				
	*fexB*	10^4.9^–10^6.2^/mL sample 10^−1.65^–10^−1.34^/16S				

**Table 4:** Abundance of antibiotic resistance genes that was present in soils irrigated with treated municipal effluent or with livestock manure. Abundances were determined by quantitative PCR.

Type of ecosystem studied	Gene class	Type of antibiotics the gene class was resistant to	Abundance	Geographical location	Ref.
7d soils subjected to one time batch irrigation, treated municipal effluent	*sul-I*	Sulfonamide	10^1.85^–10^3^/g sample	Western USA	[48]
	*sul-II*		10^3^–10^3.7^/g sample		
	*tetO*	Tetracycline	10^1.6^–10^1.7^/g sample		
	*tetW*		10^1.3^–10^1.7^/g sample		
7d soils subjected to periodic irrigation, treated municipal effluent	*sul-I*	Sulfonamide	10^2.3^–10^2.95^/g sample		
	*sul-II*		10^1.48^–10^3^/g sample		
	*tetO*	Tetracycline	10^0.95^–10^1.48^/g sample		
	*tetW*		10^1.48^–10^3^/g sample		
Irrigated soil, subjected to treated municipal wastewater irrigation	*sul-I*	Sulfonamide	ND–10^6.48^/g sample ND–10^−2.3^/16S	Shafdan, Israel	[45]
	*sul-II*		ND–10^5.3^/g sample ND–10^−3.52^/16S		
	*ermB*	Erythromycin	ND–10^3.95^/g sample ND–10^−5^/16S		
	*ermF*		ND–10^5.85^/g sample ND–10^−3.05^/16S		
	*tetO*	Tetracycline	ND		
Aquaculture, sediments	*sul-I*	Sulfonamide	10^−4.52^–10^−3.48^/16S	Tianjin, China	[86]
	*sul-II*		10^−3.7^–10^−2.74^/16S		
	*tetW*	Tetracycline	10^−4.96^–10^−3.36^/16S		
	*tetM*		ND–10^−3.7^/16S		
	*tetO*		ND–10^−4^/16S		
	*tetT*		10^−6.67^–10^−5.51^/16S		
Swine feedlot, soil	*tetM*	Tetracycline	10^−4.5^–10^−1.4^/16S	Beijing, Tianjin, Jiaxing, China	[87]
	*tetO*		10^−4.4^–10^−2.2^/16S		
	*tetQ*		10^−4.2^–10^−2^/16S		
	*tetW*		10^−4.8^–10^−2.2^/16S		
	*tetT*		ND–10^−3.2^/16S		
Swine, compost manure	*sul-I*	Sulfonamide	10^−1^/16S	New Territories, Hong Kong	[88]
	*sul-II*		10^−1.05^/16S		
	*dfrA1*		10^−1.96^/16S		
	*dfrA7*		10^−2.15^/16S		
	*tetC*	Tetracycline	10^−2.51^/16S		
	*tetG*		10^−1.72^/16S		
	*tetQ*		10^−3.4^/16S		
	*tetZ*		10^−2.52^/16S		
	*tetW*		10^−3.62^/16S		
	*tetY*		10^−1.74^/16S		
	*gyrA*	Fluoroquinolone	10^−6^/16S		
	*parC*		10^−6.35^/16S		
Swine, manure-applied soil	*tetQ*	Tetracycline	ND–10^−2.03^/16S ND–10^4.53^/ng DNA	Illinois, USA	[77]
	*tetZ*		ND–10^−3.39^/16S ND–10^2.2^/ng DNA		
Swine, manure-applied soil	*cmlA*	Chloramphenicol	10^4.69^–10^5.52^/g sample 10^−2.28^–10^−1.62^/16S	Beijing, China	[74]
	*floR*		10^4.91^–10^5.47^/g sample 10^−2.17^–10^−1.82^/16S		
	*fexA*		10^4.88^–10^5.42^/g sample 10^−2.34^–10^−1.65^/16S		
	*cfr*		10^5.01^–10^5.69^/g sample 10^−2.02^–10^−1.5^/16S		
	*fexB*		10^5.06^–10^5.61^/g sample 10^−2.03^–10^−1.61^/16S		

Furthermore, as the lagoon treatment does not ensure a total removal of antibiotic resistance genes, a substantial amount of these genes are still present when the stabilized manure is applied onto the agricultural soils. A collation of published studies suggested that manure-applied soils still contained up to 10^−1.4^ copies of tetracycline resistance genes per copy of 16S rRNA gene (Table 4). Genes that conferred resistance to sulfonamide, fluoroquinolone and chloramphenicol were also present, and were detected at abundance of up to 10^−1^ copies per copy of 16S rRNA genes. In certain instances, the abundance of associated antibiotic resistance genes in the soils increased by at least six-fold after manure application [77], indicating a transfer of resistance genes from the manure to the soil environment. An estimation from Table 1, Table 3, Table 4 suggested that the average abundance of tetracycline resistance genes and sulfonamide resistance genes from the lagoon effluent can be up to 10^−2.03^ and 10^−0.95^ copies per 16S rRNA genes, respectively. In comparison, the average abundances of tetracycline- and sulfonamide resistance genes in the treated municipal effluent were 10^−2.24^ and 10^−1.03^ copies per 16S rRNA genes, respectively. These estimates showed that the treated municipal effluent had a lower abundance of resistance genes than stabilized lagoon effluent, and may be more suitable for agricultural irrigation. For example, the average abundance of tetracycline resistance genes and sulfonamide resistance genes in the background manure-applied soil is approximately 10^−2.16^ and 10^−1.46^ copies per 16S rRNA genes, respectively, while tetracycline- and sulfonamide resistance genes in the background reclaimed water irrigated soil is below detection limits and 10^−2.58^ copies per 16S rRNA genes. The lower abundance of antibiotic resistance genes observed in a reclaimed water irrigated soil relative to a manure-applied soil suggest that performing irrigation with dilute, highly treated reclaimed water from domestic municipal wastewater treatment plant would impose a lower microbial risk than using stabilized manure effluent from livestock production farms.

## 6. Antibiotic Resistance Genes and Resistant Bacteria: Their Persistence and Fate

Even though reusing treated municipal effluent may impose a lower microbial risk than using stabilized livestock lagoon effluent, there still remain a cause of concern arising from their fate and persistence in the environment. Antibiotic resistance genes can be present in two forms, namely within a resistant bacteria or extracellularly as naked DNA. The fate and persistence of resistance genes would be dependent upon the ability of the bacterial host to proliferate and survive upon discharge into the environment. Akiyama and Savin found that when the municipal effluent is discharged into a river stream, the percentage abundance of *E. coli* at the immediate sampling point receiving municipal effluent (~38%) is significantly higher than that retrieved upstream (~7%). However, this abundance of resistant *E. coli* decreased at the sampling sites located at more than 640 m downstream (~18% and ~12%) from the effluent input [89]. Similarly, Negreanu and coworkers observed that antibiotic resistant bacteria that entered the irrigated soils from treated reclaimed water were not able to compete or survive in the soil environment [45]. This observation is in agreement with our study, which showed that short pulses of contamination had no detectable impact on the microbial composition of the soil microbiota [77]. Regardless, some of the resistant *E. coli* continued to persist in the sediments at a much higher concentration than that detected at the upstream sites [89], suggesting that fecal indicators such as *E. coli* continue to proliferate even after discharge. Our study also recovered viable bacterial isolates that were antibiotic resistant from manure-applied soils (Table 2). The persistence of viable resistant bacterial cells is of concern as they may transfer mobile genetic elements to the indigenous microbial populations.

Alternatively, extracellular DNA that contains mobile antibiotic resistant genes may be released into the environment from lysed microbial cells. These extracellular DNA are in turn adsorbed onto sediment matrix and natural organic matter that are prevalent in the soil and water environment. Natural transformation rates are dependent upon the available amount of extracellular DNA after adsorption onto surfaces such as the silica sediments. When adsorbed onto natural organic matter, the free DNA was more resistant against DNase I degradation than the free DNA [90,91,92,93]. Contradictory transformation frequencies were shown in different studies, where Crecchio and coworkers observed that bound DNA was only capable of transforming *Bacillus subtilis* at a lower frequency than free DNA [90]. Lu *et al.* however observed a 10-fold increment in the transformation frequencies for DNA adsorbed onto natural organic matter compared to dissolved DNA into competent *Azotobacter vinelandii* [94]. Regardless, both studies agreed that adsorbed DNA remains available for transformation into competent bacterial cells.

Besides adsorption onto sediments, antibiotic resistant genes associated with extracellular DNA can also be inactivated by sunlight. Antibiotic resistance genes present in soil and underwater are exposed to the ultraviolet (UV) and visible light spectrum (320–700 nm) from the sunlight. To the best of our knowledge, little is known about the inactivation kinetics of antibiotic resistance genes upon exposure to sunlight, and this knowledge gap would require more in-depth and systematic future studies. Recent studies have looked into the use of UV to dimerize antibiotic resistance genes, with the intention of first inactivating these genes prior to their discharge [95]. The findings revealed that this would require UV doses that are at least 1 order of magnitude higher than that for inactivation of the associated host bacterial cells. Generally, about 200–400 mJ/cm^2^ of UV dosage is required to result in 3–4 log damage to antibiotic resistance genes. This is approximately equivalent to the highest recommended UV dose of 186 mJ/cm^2^ to achieve 4 log removal and/or inactivation of viruses [96]. However, certain antibiotic resistance genes like *tetA* and *ampC*, which are generally present in higher abundance than *mecA* and *vanA* (Table 1), were significantly harder to inactivate. To illustrate, a UV dose of 186 mJ/cm^2^ would only achieve an inactivation of 1–2 log for *tetA* and *ampC*, while the same dose would have inactivated *mecA* and *vanA* by 3–4 log [95].

Given these observations, it would be likely that antibiotic resistance genes would continue to persist in the environment for a period of time. The antibiotic resistance genes remained elevated in their abundances and were still detectable by quantitative PCR even after 16 months of environmental exposure [77]. A similar observation was made on the abundance of *tetC*, *tetG*, *tetQ*, *tetZ* resistance genes relative to the 16S rRNA genes in swine manure compost, where these resistance genes continued to persist for up to two weeks [88]. To the best of our knowledge, limited studies are available to mechanistically determine the decay kinetics of antibiotic resistance genes under various environmental conditions (e.g., sunlight irradiation, temperature, natural organic matter, and so on). Little is also known on the factors that account for the differences in the duration of persistence among the varying types of antibiotic resistance genes. Current efforts mainly emphasized on determining the half-lives of fecal indicators like total coliforms, fecal coliforms, *Enterobacteriaceae*, Enterococci and host-specific Bacteroidales [97,98,99]. Currently, it is unknown if the same bacterial hosts which possess antibiotic resistant genes would exhibit a similar inactivation rate as the non-resistant counterparts. More studies would have to be conducted to address this knowledge gap.

## 7. “Perfect Microbial Storm”

Clearly, the rampant consumption of antibiotics by humans and animals would mean that antibiotics residues, antibiotic resistant bacteria and their associated resistance genes would be disseminated into the environment, particularly when insufficient treatment procedures are put in place to eliminate these contaminants. These microbial contaminants would be a cause of concern when performing water reuse. Some questions to ask would include: are the antibiotic resistant bacteria associated with mobile genetic elements? Are agricultural crops able to take up antibiotics residues or antibiotic resistance genes from the manure-applied soil? Do antibiotic resistance genes and resistant bacteria enter into the human food chain? Is it detrimental to health if one were to consume the contaminated crops? Providing credible information to address these questions would allow one to assess the extent of microbial risk associated with using reclaimed water—a key to success for wastewater reuse programs.

Mobile genetic elements play an important role in the dissemination of antibiotics resistance genes from one bacterium to another. The mobile genetic elements of concern would include plasmids, integron gene cassettes and transposons. To illustrate, IncP-1 plasmids are broad-host-range plasmids which are able to transfer to and replicate in phylogenetically distinct hosts. These hosts can range from gram-negative bacteria like enteric species to gram-positive bacteria and yeast. Based on the IncP plasmids that was identified and studied thus far, IncP plasmids can be grouped into five branches, namely IncP-1α, IncP-1β, IncP-1δ, IncP-1γ and IncP-1ε. Among them, IncP-1α plasmids are important for the dissemination of antibiotic resistance genes [100]. Thus far, six IncP-1α plasmids, namely pB5, pB11, pSP21, RK2, pTB11 and pBS228 were isolated from wastewaters. The plasmids were further sequenced for their genomes (Genbank accession number CP002151, CP002152, CP002153, BN000925, AJ744860, AM261760), revealing two hot spots for integration of accessory elements like heavy metal or antibiotic resistance genes (e.g., *tetAR* against tetracycline, *oxa2* against beta-lactamase, *aacA4* and *aadA1* against aminoglycoside) [100,101,102,103].

Many more new non-IncP plasmids were also recently identified in wastewater, and genomic sequences of IncN plasmids pRSB201, pRSB203, pRSB205 and pRSB206 (Genbank accession number JN102341 to JN102344) further suggesting that like IncP-1α plasmids, the four IncN plasmids isolated from wastewater also contained a variety of transposons, insertion sequences and integron gene cassettes [104]. Other studies have likewise reported an equally high abundance of mobile genetic elements (e.g., transposase, integrase genes) alongside the antibiotic resistance genes [77,105]. These observations further reiterated the need for proper treatment of reclaimed water and manure before practicing water reuse or manure application, respectively. Failure to do so would lead to the dissemination of mobile genetic elements, which would in turn become very efficient vehicles in the horizontal dissemination of antibiotic resistance genes between pathogens and the bacteria originating from wastewater and manure.

In a recent study, Forsberg and coworkers compared the extent of similarity for resistance genes among four resistance fragments in the metagenome of 95 soil-derived bacterial cultures to five human pathogenic isolates [106]. It was previously hypothesized that perfect nucleotide identity between full-length resistance genes from distinct species would imply that recent horizontal gene transfer has occurred [24,107]. Their analysis revealed that up to 99% of the contigs was identical to sequences found in numerous pathogens. The contigs also contained a series of resistance genes (e.g., resistance to aminoglycosides, tetracyclines, amphenicols and sulfonamides) that were flanked by integrase gene class 1 and transpososase [106]. Furthermore, one gene contig from the metagenome of 95 soil-derived bacterial cultures contained perfect matches to sets of resistance genes from two cultured intestinal isolates, indicating potential interconnections between the resistomes of soil isolates, clinical pathogens and enteric microorganisms [106,108].

Recognizing that unmonitored use of antibiotics would lead to increased antimicrobial resistance, the European Union (EU) banned avoparcin (*i.e.*, a glycopeptide antibiotic effective against gram-positives bacteria and shares chemical similarity to vancomycin) for all uses in agriculture in 1997. In 1999, the ban was further extended to the use of other drug classes such as tylosin, spiramycin, virginiamycin and bacitracin for growth promotion, and eventually all other antimicrobials were phased out in 2006. There remains an ongoing debate to extend the ban to antibiotics as prophylaxis [109]. After the ban on antibiotics for growth promotion, mixed results were achieved among the different EU countries. For instance, Denmark saw a reduction in vancomycin-resistant *Enterococcus faecium* first from the broilers in 1999 [110], and subsequently, a reduction in both vancomycin- and macrolide- resistant *E. faecium* from the pigs after macrolide antibiotics were banned in 1999 [111]. In the Netherlands, a discontinued use of antibiotics for growth promotion was not immediately accompanied by appropriate monitoring and disease control measures in the agricultural sector. This led to a corresponding increase in the use of antibiotics for disease treatment, to balance feed quality in broilers, and to treat non-infectious conditions such as dysbacteriosis [109]. This may be a reason to account for a continued high prevalence of fluoroquinolone-resistant *Campylobacter* and extended spectrum beta-lactamase (EBSL) producing *E. coli* and *Salmonella* in poultry and broilers, respectively [112].

Furthermore, although many types of the antibiotics were not used in the production process in Sweden, EBSL and transferable *ampC* beta lactamase-producing *E. coli* were positively detected on 44% of the tested chicken meat in Sweden. It is likely that the resistance genes may be vertically introduced into the domestic stock from imported breeding stocks that were treated with antibiotics [113]. Alternatively, Zhu and co-workers found that the resistance genes detected in the compost and soil samples were not limited to the antibiotics administered, suggesting that co-selection of resistance genes can occur in some ecosystems [105]. Transmission of these antibiotic resistant bacteria, particularly those that are foodborne pathogens like *Campylobacter*, would be of concern. An epidemiological study conducted among Dutch patients observed that 39% of the ESBL-producing *E. coli* isolated from retail meat samples was also present in humans, suggesting transmission from poultry to humans through food chains. Such antimicrobial-resistant strains would cause more prolonged or severe illness than do antimicrobial-susceptible strains [114].

Similar to the use of veterinary medicine in agriculture, unnecessary use of antibiotics in the human community can lead to increased antimicrobial resistance in wastewater. Although guidelines have been drawn to provide good guidance to doctors on proper prescription of antibiotics, it is hard to ensure that the general public would practice prudent use of antibiotics, particularly antibiotics that do not require a prescription. This would be a cause of concern, especially considering that studies have shown that plants can uptake in antibiotics residues [115,116,117] that are introduced to plants via irrigation [118]. To illustrate this, lettuces that were grown in manure-applied soil were detected with florfenical, levamisole and trimethoprim in their leaves, while carrot roots were detected with enrofloxacin, florfenicol and trimethoprim [117]. Since both lettuce and carrots are often consumed raw, human consumers are inadvertently exposed to sub-therapeutic level of antibiotics through their diet. 

A long term consumption of sub-therapeutic dosage of antibiotics may lead to enrichment of antibiotic resistance among the enteric microorganisms. Alternatively, antibiotic resistant bacteria that are consumed in raw food may also be able to reach intestinal tract and further exchange genes with the human commensal gut microbiota. For example, Salyers and coworkers focused on *Bacteroides* spp., the predominant gut bacterial population, and analyzed *Bacteroides* spp. strains obtained pre- and post-1970. They found that *ermG* and *ermB* genes (*i.e.*, erythromycin resistance genes) appeared to have entered *Bacteroides* species from some other bacterial species as the pre-1970 *Bacteroides* strains did not carry these genes. Instead, *erm* genes were previously exclusively associated with gram-positive bacteria, including *Streptococcus pneumonia* and *Clostridium perfringens*, but had in recent years, horizontally moved between gram-positive bacteria and enteric *Bacteroides*. Similarly, *tetM* (*i.e.*, tetracycline resistance genes) can also be transferred between *Campylobacter* and other enteric microorganisms like Clostridia and *Bifidobacterium* spp. [24]. It is therefore likely that the gut may also function as a hotspot for horizontal transfer of resistance genes from contaminated food and water supply, to the enteric gut bacterial populations. However, providing evidence to justify this hypothesis would be challenging as it would be hard to determine the direction of horizontal gene transfer. Also, transfer frequencies may be low and hard to detect. Furthermore, little is known about the dose of antibiotics resistant bacteria or antibiotics residues required to cause selection of resistance genes or transfer of antibiotic resistance genes to gut microbiota [24]. More systematic studies would have to be conducted to assess the actual extent of microbial risk and the scale of antimicrobial resistance issues.

## 8. Conclusions

To conclude, reclaimed water is a promising alternative water source to address the global water scarcity issue. However, antibiotics residues can select for antibiotic resistant bacteria that harbor mobile genetic elements. The mobile genetic elements can in turn be associated with resistance genes that are transferred to other indigenous microorganisms in the water, soil and gut ecosystem. Given the potential risks, a concerted effort of good management strategy, extensive monitoring and cost-effective engineering treatment processes must be put in place before one can reap the benefits of adopting water reuse from reclaimed water.
